# Mapping the Evidence on Oral Health Interventions and Cognitive Status in Alzheimer’s Disease: A Scoping Review

**DOI:** 10.3390/brainsci16060615

**Published:** 2026-06-07

**Authors:** Man Hung, Hanna Chriss, Megan Nelson, Janaki O’Callaghan, Corban Ward, Alicia Parry, Jacob Marx, Martin S. Lipsky

**Affiliations:** 1 College of Dental Medicine, Roseman University of Health Sciences, South Jordan, UT 84095, USA; 2 Department of Family Medicine and Public Health, University of Utah, Salt Lake City, UT 84108, USA; 3 College of Education, University of Utah, Salt Lake City, UT 84112, USA; 4 VA Salt Lake City Health Care, Salt Lake City, UT 84148, USA; 5 Library, Roseman University of Health Sciences, South Jordan, UT 84095, USA; 6 Institute on Aging, Portland State University, Portland, OR 97207, USA

**Keywords:** Alzheimer’s disease, oral health, cognition, periodontal disease, oral microbiome

## Abstract

**Highlights:**

**What are the main findings?**

- Evidence consistently links oral health status with cognitive outcomes in Alzheimer’s disease.
- Interventional studies are limited and do not provide clear evidence of cognitive improvement.

**What are the implications of the main findings?**

- Current evidence is insufficient to establish oral health as a causal or therapeutic target for improving cognition.
- Future research should integrate standardized cognitive measures with mechanistic outcomes to clarify pathways.

**Abstract:**

Background: Oral health is increasingly recognized as a potentially modifiable factor in Alzheimer’s disease (AD), although its influence on cognitive outcomes remains uncertain. Methods: This scoping review followed the Arksey and O’Malley framework and was reported in accordance with PRISMA-ScR guidelines. Searches were conducted in PubMed, Scopus, and Web of Science through August 2025. Eleven studies met the inclusion criteria: one randomized controlled trial, one nonrandomized trial, and nine observational studies. Results: Poor oral health, including tooth loss, periodontal disease, and impaired mastication, was consistently associated with worse cognitive and dementia-related outcomes. Interventions improved oral and functional measures but yielded limited and inconsistent evidence of cognitive benefit. Proposed mechanisms, including systemic inflammation and microbiome alterations, were infrequently evaluated directly. Conclusions: Overall, oral health correlates with cognitive status in AD, but the causal impact of interventions remains uncertain, highlighting the need for rigorous trials with standardized cognitive and mechanistic outcomes.

## 1. Introduction

Alzheimer’s disease (AD) is a progressive neurodegenerative disorder and the leading cause of dementia worldwide [1]. It is characterized by cognitive and functional decline, imposing substantial psychological, social, and economic burdens on patients, families, and health care systems. With global population aging, the prevalence of AD is projected to exceed 150 million by 2050 [2]. Despite advances in understanding disease mechanisms, effective disease-modifying therapies remain limited, highlighting the importance of identifying potentially modifiable risk factors and supportive nonpharmacological strategies.

Oral health has emerged as a potentially important factor in this context. Poor oral health, including periodontal disease, tooth loss, impaired mastication, and oral microbiome dysbiosis, is associated with cognitive impairment and dementia [3,4]. Proposed mechanisms include systemic inflammation, microbial translocation, and vascular and immune dysregulation. For example, *Porphyromonas gingivalis* has been detected in the brains of individuals with AD, and its virulence factors have been implicated in amyloid-β and tau pathology [5]. Together, these findings support the concept of a “mouth–brain axis” linking oral disease and neurodegenerative processes.

Despite growing epidemiological and mechanistic evidence, it remains unclear whether oral health represents a modifiable determinant of cognitive outcomes in AD or primarily reflects downstream consequences of disease progression. Studies evaluating oral health interventions, including professional dental care, caregiver-assisted oral hygiene programs, antimicrobial treatments, and prosthodontic rehabilitation, remain limited and methodologically variable [6,7]. Recent reviews have similarly noted inconsistent outcome assessment, short follow-up periods, and limited integration of mechanistic biomarkers with longitudinal cognitive outcomes [8,9]. In addition, much of the current evidence is observational or cross-sectional, limiting causal inference and interpretation [10].

Previous reviews have largely focused on oral health and dementia risk, with greater emphasis on observational associations than interventional evidence [8]. Few reviews integrate clinical interventions, mechanistic pathways, caregiver-supported oral care, and implementation-related considerations within AD populations.

Accordingly, this scoping review aimed to systematically map and synthesize evidence on oral health interventions and oral health-related exposures in individuals with AD. Specifically, we evaluated (1) oral health interventions and exposures studied in AD populations, (2) cognitive and dementia-related outcomes, (3) proposed biological mechanisms linking oral health and AD, and (4) major methodological limitations and knowledge gaps. Importantly, this review examined whether oral health may represent a potentially modifiable factor influencing cognitive outcomes in AD or instead reflect consequences of progressive neurocognitive decline.

## 2. Methods

### 2.1. Study Design and Framework

This scoping review followed the methodological framework proposed by Arksey and O’Malley and further refined by Levac et al. and the Joanna Briggs Institute (JBI) for scoping reviews [11]. The review reported its results in accordance with the Preferred Reporting Items for Systematic Reviews and Meta-Analyses Extension for Scoping Reviews (PRISMA-ScR) guidelines to ensure transparency and methodological rigor [12]. It was registered on the Open Science Framework (OSF; Registration DOI: 10.17605/OSF.IO/4QYGH). 

A scoping review approach was selected because of the heterogeneity of study designs, the emerging nature of the field, and the need to map both interventional and observational evidence. The objective of this review was to comprehensively characterize the existing literature on oral health-related interventions and oral health-related exposures in individuals with AD, with particular emphasis on cognitive outcomes, proposed biological mechanisms, and implementation considerations.

### 2.2. Eligibility Criteria

Eligibility criteria were defined using the Population–Concept–Context framework recommended by the JBI.

Population: Studies were eligible if they included adults diagnosed with AD using established clinical or research criteria (e.g., NINCDS–ADRDA, DSM-5, ICD-10, or equivalent). Studies involving participants with mixed dementia or mild cognitive impairment (MCI) were included if data specific to individuals with AD were reported separately or if AD represented the predominant diagnosis within the study population.

Concept: The concept of interest encompassed both (1) oral health interventions and (2) oral health-related exposures or conditions. Oral health interventions were defined as strategies aimed at improving oral hygiene, periodontal status, oral function, or oral microbial balance (e.g., professional dental care, caregiver-assisted oral hygiene, antimicrobial therapies, prosthodontic rehabilitation, oral-motor exercises, and oral health education). Oral health-related exposures included, but were not limited to, periodontal disease, tooth loss, denture status, masticatory function, salivary parameters, and oral microbiome characteristics. To be included, studies were required to report at least one cognitive or dementia-related measure, including global cognitive assessments (e.g., Mini-Mental State Examination [MMSE], Montreal Cognitive Assessment [MoCA]), validated dementia staging scales (e.g., Clinical Dementia Rating [CDR], Functional Assessment Staging Tool [FAST]), or Alzheimer-specific cognitive instruments (e.g., ADAS-Cog). Studies in which cognitive measures were used solely for diagnostic classification or staging were included if they contributed to understanding the relationship between oral health and cognitive status. Studies reporting biological or mechanistic outcomes (e.g., inflammatory markers, oral microbiota, salivary biomarkers, or neuroimaging findings) and/or implementation-related outcomes (e.g., feasibility, adherence, and caregiver involvement) were also eligible.

Context: All care settings were eligible, including long-term care facilities, outpatient memory clinics, dental clinics, community-based settings, and home care environments.

Study Design: A broad range of study designs was included, consistent with scoping review methodology, including randomized controlled trials, nonrandomized trials, quasi-experimental studies, longitudinal cohort studies, and cross-sectional or case–control observational studies. No minimum sample size cutoff was applied because the objective of this scoping review was to comprehensively map the available literature, including exploratory and pilot studies relevant to oral health and AD.

Exclusion Criteria: We excluded review articles (e.g., systematic reviews, meta-analyses, and narrative reviews), editorials, commentaries, conference abstracts, nonhuman studies, and studies not published in English or not reporting primary data relevant to the review objectives (Table 1).

### 2.3. Information Sources and Search Strategy

A comprehensive search strategy was developed in consultation with a health sciences librarian. Electronic database searches were conducted in PubMed, Scopus, and Web of Science from January 2015 through August 2025. The authors selected the 2015 cutoff to align more closely with major developments in biological concepts and diagnostic criteria for AD over the past decade, as well as growing recognition of the potential role of the oral–brain axis in cognitive decline and neurodegenerative disease.

The search strategy combined controlled vocabulary (e.g., Medical Subject Headings [MeSH] in PubMed) and free-text keywords related to AD and oral health. Search terms included variations of “Alzheimer disease,” “oral health,” “oral hygiene,” and “dental care,” along with terms capturing both interventional and observational concepts. Boolean operators (AND/OR), truncation, and database-specific syntax were used to optimize sensitivity and specificity. The full database-specific search strategies are provided in Table 2.

### 2.4. Study Selection

**Figure 1:**
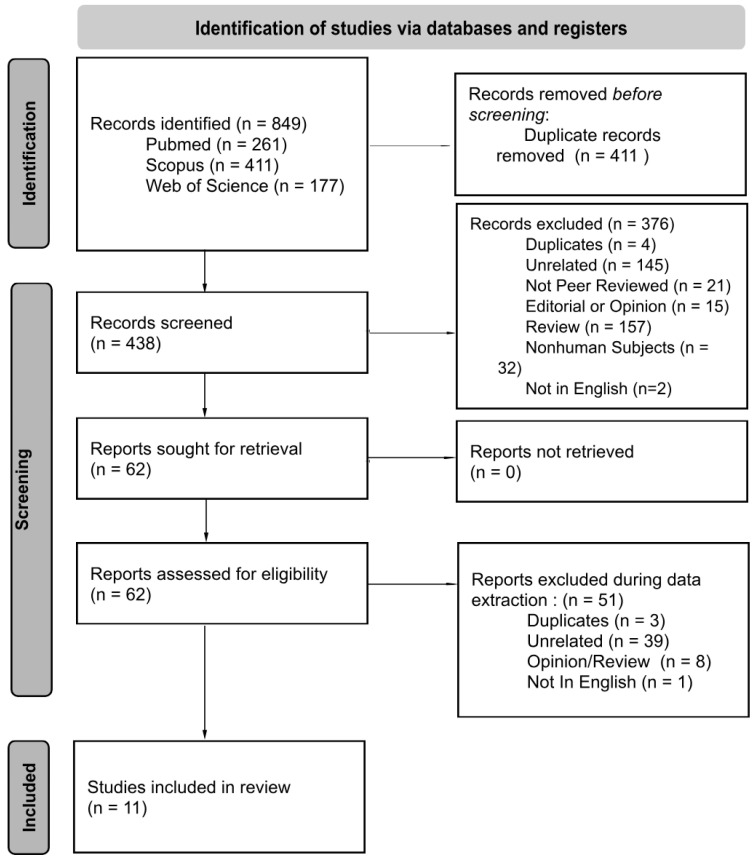
PRISMA flow chart.

All records identified through the search were imported into EndNote Version 21 (Clarivate Analytics, Philadelphia, PA) for de-duplication [11]. Title and abstract screening were conducted independently by three reviewers using predefined eligibility criteria. Potentially relevant articles underwent full-text review by the same reviewers. Discrepancies at both screening stages were resolved through discussion and consensus, with arbitration by an additional reviewer when necessary. The study selection process was documented using a PRISMA-ScR flow diagram, including reasons for exclusion at the full-text stage (Figure 1).

### 2.5. Data Extraction

A standardized data extraction form was developed and pilot-tested to ensure consistency and completeness. Data extraction was performed independently by three reviewers and cross-checked for accuracy.

Extracted data included (1) bibliographic information (author, year, country); (2) study characteristics (design, setting, sample size, and population demographics); (3) oral health exposure or intervention details (type, duration, frequency, and delivery model); (4) cognitive or dementia-related outcomes and measurement tools; (5) biological or mechanistic outcomes (e.g., inflammatory markers and microbiome data); (6) implementation factors (e.g., feasibility, adherence, and caregiver involvement); and (7) key findings relevant to oral health and cognitive or dementia-related outcomes.

### 2.6. Data Syntheses

Given the heterogeneity in study design, interventions, exposures, and outcome measures, quantitative synthesis was not appropriate. Instead, a structured narrative synthesis was conducted to critically evaluate patterns across studies, compare methodological approaches, and identify consistencies, discrepancies, and gaps in the evidence.

Studies were grouped thematically into (1) oral health interventions, (2) oral health-related exposures, (3) cognitive outcomes, (4) mechanistic findings, and (5) implementation considerations. Results were summarized in tabular format and accompanied by a structured narrative synthesis integrating findings across domains.

Particular attention was given to evaluating methodological limitations across studies, including study design, participant characteristics, exposure and outcome measurement, and potential sources of bias, to inform interpretation of findings.

Formal risk-of-bias assessment and meta-analysis were not performed, consistent with scoping review methodology; however, study limitations and potential biases were considered qualitatively during data synthesis.

## 3. Results

### 3.1. Study Selection and Characteristics

The database search identified 849 records across PubMed, Scopus, and Web of Science, of which eleven studies met the inclusion criteria following screening and full-text review (Figure 1).

Included studies were published between January 2015 and August 2025 and represented diverse geographic regions, including Asia, Europe, North America, and South America. The included studies were methodologically heterogeneous and comprised one randomized controlled trial, one nonrandomized clinical trial, and nine observational studies, including cross-sectional, case–control, and retrospective cohort designs. Sample sizes varied, ranging from small clinical cohorts (n < 32) to large population-based datasets. Notably, the retrospective cohort study by Kulkarni et al. analyzed TriNetX electronic health record data from adults aged ≥60 years, including a poor oral health cohort of 1,232,751 individuals matched to comparator cohorts to evaluate subsequent AD risk [13].

Most studies included adults diagnosed with AD using established clinical or research criteria, including DSM-based diagnoses, NINCDS–ADRDA criteria, ICD codes, or validated dementia staging tools [14]. However, study populations and designs varied across the included literature. While many investigations enrolled participants already diagnosed with AD, some studies evaluated oral health as a potential risk factor for subsequent AD development in broader populations [13]. Most studies included only individuals with mild-to-moderate AD, although several studies examined participants across the disease severity continuum or evaluated dementia severity as an exposure [7].

Cognitive measures were most commonly assessed using global screening instruments, including the MMSE and MoCA, as well as staging scales such as CDR and FAST. Use of domain-specific neuropsychological assessments was limited [7]. Across the included studies, poorer oral health was consistently associated with worse cognitive, functional, or neuropsychiatric status in individuals with AD. Common findings included increased periodontal disease burden, tooth loss, impaired mastication, reduced salivary flow, altered oral microbiota, and increased dependence on caregiver-supported oral hygiene. Several studies reported statistically significant associations between oral health measures and cognitive or behavioral outcomes; however, most evidence remained observational and cross-sectional.

Table 3 summarizes the study characteristics, oral health measures, cognitive assessments, and key findings. Table 4 summarizes the clinical oral health findings, interventions, and cognitive associations, while Table 5 separately summarizes implementation barriers, caregiver-related considerations, and mechanistic domains.

### 3.2. Oral Health Interventions and Exposures

Across the eleven included studies, oral health was examined predominantly as an exposure or clinical characteristic rather than as the target of an intervention [6,14,18,20]. Observational studies evaluated a broad range of oral health-related factors, including tooth loss, periodontal disease, plaque accumulation, denture status, reduced occlusal support, impaired mastication, salivary dysfunction, and overall oral disease burden [2,17,21].

Only two studies evaluated structured oral health interventions. One nonrandomized clinical study assessed prosthodontic rehabilitation using removable partial or complete dentures in individuals with mild AD [15,17]. The second, a randomized controlled trial, evaluated a multicomponent oral health intervention incorporating structured oral care support, self-care routines, and oral health education delivered in a long-term care setting [16]. No included study evaluated intensive periodontal therapy with longitudinal cognitive follow-up using a rigorously randomized design.

In addition to direct oral health measures, several studies examined functional and nutritional correlates of oral health, including masticatory performance, dietary modifications (e.g., soft food diets), and serum albumin levels. These variables were often conceptualized as potential mediators linking oral health status to cognitive or systemic outcomes [14,21]. Additionally, several studies explored mechanistic and implementation-related domains, including oral microbiome dysbiosis, inflammatory pathways, caregiver-supported oral hygiene, resistance to oral care, and barriers to maintaining oral health in institutional or dependent-care settings.

Table 4 summarizes oral health findings, interventions, and cognitive associations. Implementation barriers, caregiver considerations, and mechanistic domains are summarized separately in Table 5.

### 3.3. Cognitive Outcomes

Across studies, cognitive measures were frequently used for diagnostic classification or disease staging rather than as primary outcomes of intervention [6,18]. Overall, the included literature consistently demonstrated associations between poorer oral health and worse cognitive or dementia-related status across diverse study populations and clinical settings. Although methodological approaches varied, observational findings suggested that greater oral disease burden was associated with more advanced cognitive impairment and functional decline. Among observational studies, poorer oral health was consistently associated with worse cognitive or dementia-related measures. Specifically, greater tooth loss, reduced occlusal support, higher periodontal burden, and impaired mastication were associated with lower MMSE or MoCA scores and more advanced dementia staging [14,20,21].

Several studies spanning different stages of AD suggested associations between compromised oral health and greater cognitive impairment [20]. However, potential confounding factors, including frailty, nutritional status, socioeconomic conditions, and access to care, were not consistently accounted for.

The single randomized controlled trial reported statistically significant improvements in global cognitive scores (MMSE) following a multicomponent oral health intervention, alongside improvements in oral health status and functional outcomes [16]. However, follow-up duration was limited, and cognitive assessment relied on global screening measures rather than domain-specific testing. The nonrandomized prosthodontic intervention study did not evaluate cognitive outcomes as a primary endpoint. Overall, evidence regarding the impact of oral health interventions on longitudinal cognitive trajectories in AD remains limited.

### 3.4. Oral Health Outcomes

All included studies reported at least one oral health-related outcome, although measurement approaches varied. Commonly assessed indicators included periodontal indices (plaque index, bleeding on probing, probing depth, and clinical attachment loss), tooth loss, denture status, occlusal support, masticatory performance, salivary flow, and oral health-related quality of life [2,6,18,20]. Tooth loss was generally assessed using clinical dental examination or tooth-count measures, whereas periodontal disease was evaluated using standard periodontal indices, including probing depth, bleeding on probing, plaque index, and clinical attachment loss. Caries prevalence and restorative status were commonly assessed using DMFT indices or clinical oral examination findings.

Across the included studies, oral health impairments were consistently reported in individuals with AD, although the specific outcomes and assessment methods varied. Overall, the evidence suggested that worsening cognitive and functional status was accompanied by poorer oral hygiene, greater periodontal burden, impaired oral function, and increased dependence on caregiver-supported oral care. Across observational studies, individuals with AD consistently demonstrated poorer oral health compared with cognitively healthy controls. Findings included a higher prevalence of periodontal disease, increased plaque accumulation, impaired mastication, and reduced salivary function [6,14,17].

In the prosthodontic intervention study, denture rehabilitation improved masticatory efficiency and oral health-related quality of life. However, masticatory performance remained significantly lower in individuals with AD compared with cognitively healthy controls, suggesting that prosthetic rehabilitation may partially restore oral function but not fully normalize it [15,17].

### 3.5. Mechanistic Outcomes

Several studies have proposed biological and functional mechanisms linking oral health and AD, although direct mechanistic evaluation was limited. Proposed pathways included systemic inflammation, oral microbial dysbiosis, impaired immune function, reduced sensory stimulation from mastication, and nutritional deficiencies [2,14,20]. Microbiome-focused studies reported reduced salivary microbial diversity and altered microbial composition in individuals with AD, including increased abundance of taxa such as *Moraxella*, *Leptotrichia*, and *Sphaerochaeta*. However, these studies did not identify specific bacteria associated with AD severity [2].

Additional hypothesized mechanisms included inflammatory signaling pathways, increased blood–brain barrier permeability, and reduced hippocampal stimulation associated with tooth loss and impaired occlusal function [15,16]. However, in most cases, mechanistic relationships were inferred rather than directly measured within the included studies [14,20]. Mechanistic outcomes were rarely assessed longitudinally or in conjunction with intervention-related cognitive changes, limiting causal interpretation.

### 3.6. Implementation Factors

Several studies reported barriers and facilitators related to the implementation of oral health care in individuals with AD. Common barriers included progressive cognitive and functional decline, leading to increased dependence on caregivers for oral hygiene [6,19]. Behavioral and psychological symptoms of dementia, such as resistance to care, agitation, and refusal behaviors, further complicated oral hygiene maintenance, particularly in moderate-to-severe stages of disease [19].

Institutional barriers included limited caregiver training, staffing constraints in long-term care settings, lack of integration between dental and medical services, and limited experience or discomfort among dental professionals in managing patients with cognitive impairment [6,14,18]. Oral hygiene was often deprioritized in institutional care environments [6,17].

Facilitators of effective oral health care included earlier disease stage, active caregiver involvement, and interdisciplinary care models [16,19]. Structured oral health programs incorporating caregiver education, routine monitoring, and multidisciplinary collaboration were identified as feasible and potentially effective approaches, particularly in long-term care settings [15,16,17].

## 4. Discussion

### 4.1. Principal Findings

This scoping review synthesizes evidence from eleven studies examining oral health-related interventions and exposures in individuals with AD and highlights a field characterized by consistent associative findings but limited interventional and mechanistic evidence. Across studies, individuals with AD consistently demonstrated poorer oral health, including higher prevalence of periodontal disease, tooth loss, impaired mastication, and reduced salivary function, compared with cognitively healthy populations or across stages of disease severity [2,17,21]. Although these findings were generally consistent across diverse populations and study designs, the predominance of observational and cross-sectional evidence limits causal inference and interpretation of directionality [15,16].

Importantly, the current evidence base reflects a fundamental imbalance: while observational associations are well documented, interventional studies testing whether modifying oral health can influence cognitive trajectories remain scarce. This imbalance limits the ability to translate epidemiologic and mechanistic insights into clinically actionable strategies.

Similarly, a recent scoping review examined the relationship between oral diseases and AD, with an emphasis on biological mechanisms, inflammatory pathways, microbial dysbiosis, and oral disease as a potential risk factor for neurodegeneration [22]. In contrast, the present review expands upon this literature by additionally evaluating oral health interventions, implementation considerations, caregiver-supported oral care strategies, and clinically oriented outcomes across diverse care settings. Furthermore, the current review distinguishes between studies using cognitive measures primarily for dementia classification or staging and those examining cognitive change as a longitudinal or interventional outcome. Together, these reviews provide complementary perspectives on the evolving relationship between oral health and AD.

### 4.2. Critical Appraisal of Interventional Evidence

Only two studies evaluated oral health interventions in AD populations, highlighting a substantial gap in the literature. The randomized controlled trial demonstrated that a multicomponent oral health intervention, combining structured oral care, caregiver-supported routines, and education, was feasible and associated with improvements in oral health, global cognitive scores, and functional outcomes [16]. However, the strength of inference is constrained by relatively short follow-up, reliance on global cognitive screening tools, and absence of mechanistic or domain-specific cognitive endpoints.

The nonrandomized prosthodontic study demonstrated improvements in masticatory function and oral health-related quality of life following denture rehabilitation but did not evaluate cognitive outcomes as a primary endpoint [15]. Residual impairment in masticatory function relative to cognitively healthy individuals further suggests that prosthetic rehabilitation alone may not fully restore functional deficits potentially relevant to neurocognitive pathways.

Collectively, the interventional evidence remains preliminary and insufficient to establish efficacy, durability of effect, or causal mechanisms. The absence of adequately powered longitudinal randomized trials with integrated biological and cognitive endpoints remains a major limitation of the field.

### 4.3. Interpretation of Observational Evidence

Observational studies consistently demonstrated associations between poor oral health and worse cognitive or dementia-related measures, including lower MMSE or MoCA scores and more advanced dementia staging [20]. However, interpretation is limited by substantial methodological differences, including variability in oral health measures, cognitive assessments, study populations, and adjustment for confounding variables. Most studies employed cross-sectional designs, limiting assessment of temporality and making it difficult to disentangle cause from consequence. Potential confounding factors, including frailty, nutritional status, socioeconomic disadvantage, comorbidity burden, and access to care, likely influence both oral health and cognitive outcomes but were not consistently controlled for across studies [13,14,20]. Reverse causality also remains plausible, as cognitive and functional decline may contribute to worsening oral hygiene through increased caregiver dependence and reduced self-care capacity [6,16,18].

In addition, the observational evidence base included heterogeneous populations. While most studies evaluated individuals already diagnosed with AD, some examined poor oral health as a potential risk factor for subsequent AD development in broader populations. For example, Kulkarni et al. reported an increased subsequent risk of AD among individuals with poor oral health [13]. Collectively, these findings support an association between oral health and AD-related outcomes but do not establish a direct causal relationship.

### 4.4. Mechanistic Integration: Bridging Biology and Clinical Evidence

A growing body of mechanistic research supports the hypothesis that oral health influences AD through biological pathways; however, the integration of these mechanisms into clinical studies remains limited.

Proposed pathways, including systemic inflammation, oral microbiome dysbiosis, microbial translocation, and vascular and immune dysregulation, are biologically plausible and supported by experimental data. Studies reporting reduced microbial diversity and altered salivary microbiome composition in individuals with AD are consistent with these hypotheses; however, current clinical evidence has not demonstrated specific oral bacterial taxa consistently associated with AD severity.

Importantly, these proposed mechanisms may explain the observed associations linking poor oral health with worse cognitive performance, greater dementia severity, impaired mastication, and functional decline. Chronic oral inflammation and microbial dysbiosis may contribute to systemic inflammatory signaling and neuroinflammation, thereby providing biologically plausible pathways linking oral disease and neurodegenerative processes.

At the same time, the relationship between oral health and AD is likely bidirectional. Progressive cognitive and functional decline in AD may impair oral hygiene behaviors, increase caregiver dependence, alter nutritional intake, and amplify inflammatory and microbiological changes, leading to deterioration of oral health over time. Together, these observations support the concept of a dynamic “mouth–brain axis” linking oral and neurocognitive health [10,23].

Nevertheless, mechanistic pathways remain inferred rather than directly demonstrated clinically because few studies simultaneously evaluated oral health, biological intermediates, and longitudinal cognitive outcomes.

### 4.5. Implementation and Health System Considerations

Beyond biological and clinical questions, the delivery of oral health care in individuals with AD presents substantial practical challenges. As disease severity increases, oral hygiene becomes increasingly dependent on caregivers, and behavioral symptoms such as agitation and resistance to care can significantly hinder intervention delivery [16]. System-level barriers, including limited caregiver training, inadequate integration of dental and medical services, and workforce constraints in long-term care settings, further limit the feasibility of sustained oral health interventions [8]. Notably, oral health is frequently deprioritized within dementia care despite its potential impact on nutrition, quality of life, and overall health outcomes.

Facilitators identified across studies, including early-stage intervention, caregiver engagement, and structured care protocols, suggest that successful implementation will depend not only on intervention efficacy but also on integration into existing care systems. Multicomponent, caregiver-supported models appear particularly promising but require further evaluation in pragmatic, real-world settings.

### 4.6. Strengths and Limitations of the Evidence Base

This review has several important strengths. To our knowledge, it is among the few scoping reviews specifically synthesizing evidence on oral health, oral health interventions, and cognitive outcomes in individuals with AD across clinical, functional, and mechanistic domains. The review incorporated studies from diverse geographic regions and healthcare settings and included a broad range of oral health exposures, interventions, and biological outcomes. In addition, the scoping review methodology allowed comprehensive mapping of an emerging and methodologically heterogeneous field while identifying major knowledge gaps and priorities for future research.

Methodological limitations should be considered when interpreting the current evidence base. Substantial clinical and methodological heterogeneity existed across studies, including differences in study design, participant characteristics, oral health measures, cognitive assessments, and outcome definitions. Most included studies were observational and cross-sectional, limiting the ability to establish temporality or causality and raising the possibility of reverse causation.

Adjustment for important confounding variables, including frailty, education, socioeconomic status, nutritional status, comorbidity burden, and access to dental care, was inconsistent across studies. Most investigations also relied primarily on global cognitive screening instruments such as the MMSE and MoCA rather than domain-specific neuropsychological assessments, potentially limiting sensitivity to subtle cognitive changes in memory, executive function, attention, or processing speed. Mechanistic biomarkers, including inflammatory and microbiological measures, were rarely integrated with longitudinal clinical outcomes, restricting evaluation of proposed biological pathways linking oral health and AD. Consistent with scoping review methodology, a formal risk-of-bias assessment was not performed; therefore, the findings should be interpreted as a broad synthesis of the available literature rather than a measure of evidence quality or certainty.

Finally, the search strategy intentionally prioritized broad oral health concepts specific to AD rather than exhaustive inclusion of individual oral health subdomains or broader dementia terminology, which may have led to the omission of potentially relevant studies. Collectively, these limitations highlight the need for larger longitudinal and interventional studies using standardized oral health measures, domain-specific cognitive assessments, and integrated mechanistic biomarkers.

### 4.7. Research Priorities and Testable Hypotheses

Advancing this field will require a shift toward hypothesis-driven, mechanistically informed interventional research. Future studies should prioritize adequately powered randomized controlled trials with longer follow-up periods and standardized assessments of both oral health and cognition.

Importantly, testable hypotheses emerge from the current evidence base. First, improving periodontal health may reduce systemic inflammatory burden and, in turn, attenuate cognitive decline in early-stage AD. Second, restoration of masticatory function may improve nutritional status and sensory input, potentially stabilizing cognitive performance. Third, targeted modulation of the oral microbiome may influence neuroinflammatory pathways implicated in AD progression. Testing these hypotheses will require integrated study designs incorporating clinical, biological, and behavioral endpoints, as well as careful consideration of disease stage and care context.

### 4.8. Implications for Practice and Policy

Although causality remains unproven, the consistent association between poor oral health and adverse outcomes in AD suggests that oral health represents a potentially modifiable component of dementia care. Routine oral health assessment, caregiver training, and integration of dental services into dementia care pathways may improve quality of life and overall health outcomes, even in the absence of demonstrated cognitive benefits. At the policy level, greater recognition of oral health within multidisciplinary dementia care frameworks may facilitate more comprehensive and patient-centered approaches. However, widespread implementation should be guided by evidence from rigorously designed trials.

## 5. Conclusions

This review demonstrates that poor oral health is consistently associated with worse cognitive and dementia-related outcomes in individuals with AD, whereas evidence supporting the effectiveness of oral health interventions remains limited. Current findings support the biological plausibility and clinical relevance of the oral health–cognition relationship but do not establish causality. Addressing this gap will require rigorous interventional and mechanistic studies designed to determine whether oral health represents a viable target for modifying disease progression in AD.

## Figures and Tables

**Table 1:** Inclusion and exclusion criteria.

Inclusion Criteria	Exclusion Criteria
Peer-reviewed primary research articles	Review articles (systematic reviews, meta-analyses, scoping reviews, and narrative reviews)
Studies published between January 2015 and August 2025	Editorials, commentaries, letters to the editor, and opinion pieces
Studies involving adults diagnosed with AD using clinical or research criteria (e.g., DSM-5, NINCDS–ADRDA, ICD-based diagnoses)	Conference abstracts or proceedings without full-text articles
Studies evaluating oral health interventions (e.g., oral hygiene programs, periodontal therapy, prosthodontic rehabilitation, oral health education) or examining oral health-related exposures or conditions (e.g., periodontal disease, tooth loss, denture status, masticatory function, oral microbiome)	Studies not focused on AD or not reporting AD-specific data
Studies reporting cognitive outcomes, dementia-related measures (e.g., staging or severity), or oral/biological outcomes relevant to AD	Studies not published in English
All study designs, including randomized controlled trials, nonrandomized trials, cohort studies, case–control studies, and cross-sectional studies	Non-human (animal or in vitro) studies
Full-text articles available	Studies with insufficient data for extraction

**Table 2:** Search strategy.

Database (Date of Search)	Search Strategy	Number of Records Found
PubMed (8 June 2025)	((“oral intervention”[tiab]) OR (“oral hygiene”[tiab]) OR (“Oral health”[Title/Abstract]) OR (“dental care”[tiab]) OR (“dental intervention”[tiab]) OR (“dental hygiene”[tiab]) OR (“periodontal therapy”[tiab]) OR (Periodontal treatment[Title/Abstract]) OR (Periodontal intervention[Title/Abstract])) AND ((“Alzheimer Disease”[Mesh]) OR (“alzheimer”[tiab]) OR (“AD”[tiab]))	261
Scopus (8 June 2025)	(TITLE-ABS-KEY (“Oral intervention” OR “Oral hygiene” OR “Oral Health” OR “Dental care” OR “Dental Intervention” OR “Dental hygiene” OR “Periodontal therapy” OR “Periodontal treatment” OR “Periodontal intervention”)) AND (TITLE-ABS-KEY (“Alzheimer Disease” OR “alzheimer” OR “AD”))	411
Web of Science (8 June 2025)	AB = (((“oral intervention”) OR (“oral hygiene”) OR (“Oral health”) OR (“dental care”) OR (“dental intervention”) OR (“dental hygiene”) OR (“periodontal therapy”) OR (“Periodontal treatment”) OR (“Periodontal intervention”)) AND ((“Alzheimer Disease”) OR (“alzheimer”) OR (“AD”)))	177

**Table 3:** Characteristics and key findings of included studies.

Author (Year)	Country	Study Design	Setting and Population	Sample Size	AD Criteria/Stage	Oral Health Variables	Cognitive Measures	Key Findings
* Aragon (2018) [6]	Spain	Case–control study	Alzheimer Center Reina Sofía and State Reference Center; adults aged ~60–99 years	106	Mild to Severe	DMFT/DMFS, CPI, salivary flow, prostheses, oral hygiene	FAST, CDR, GDS	AD patients had fewer teeth, worse periodontal status, lower salivary flow, and higher candidiasis prevalence than controls.
Campos (2018) [15]	Brazil	Nonrandomized clinical trial	Piracicaba Dental School; adults (mean age ~75 years)	32	Mild	Denture rehabilitation, masticatory efficiency, salivary flow	MMSE, CDR	Prosthodontic rehabilitation improved masticatory efficiency and oral health-related quality of life.
Chen (2022) [16]	China	Randomized control trial	Long-term care center; adults ≥60 years	66	Mild	BOHSE, oral microbiota	MMSE, NPI, NHAS, ADCS-ADL	After 24 weeks, the intervention group demonstrated significantly improved BOHSE, MMSE, NPI, NHAS, and ADCS-ADL scores (*p* < 0.05) and healthier oral microbiota composition.
Frota (2016) [17]	Brazil	Cross-sectional observational study	Geriatric centers; adults aged 57–91 years	90	Mild to Severe	Caries, periodontal disease, denture status, denture stomatitis	MMSE used diagnostically	54.2% of AD patients had periodontal disease, and 34.2% had caries; denture stomatitis was significantly higher in the dementia group (*p* = 0.0213).
†* Kulkarni (2023) [13]	United States	Retrospective cohort study	TriNetX database; adults ≥60 years with poor oral health versus normal oral health evaluated for subsequent AD risk using matched cohorts	31,418,814 normal oral health cohort; 1,232,751 poor oral health cohort	Not reported	Tooth loss, periodontal disease, caries, gingivitis	ICD-coded AD diagnosis	Poor oral health was associated with >2-fold increased AD risk (RR 2.363; 95% CI 2.326–2.401); tooth-loss-related disease showed the highest risk (RR 3.186; 95% CI 3.007–3.376).
†* Laugisch (2021) [18]	Germany	Cross-sectional case–control study	University of Münster memory clinic; adults aged 30–65 years with AD and other dementia subtypes	40	Mild to Moderate	CAL, PPD, BOP, plaque, radiographic bone loss	MMSE, CSF biomarkers	Periodontitis was identified in all participants; no significant periodontal differences were found between AD and non-AD dementia groups.
†* Liu (2019) [2]	China	Matched case–control study	Xiangya Hospital; adults with AD and cognitively healthy controls (mean age ~64 years)	78 (39 AD, 39 controls)	Mild to Severe	Salivary microbiome diversity and composition	MMSE, CDR, ADL	AD patients demonstrated lower microbial diversity and altered salivary taxa; no bacteria were associated with disease severity.
Sherbaf (2025) [14]	Hungary	Cross-sectional observational study	University of Szeged; adults aged 66–97 years	81	Mild to Moderate	Plaque index, probing depth, attachment loss	MMSE	Worse periodontal parameters were associated with AD severity.
Shirobe (2023) [19]	Japan	Cross-sectional observational Study	Long-term care facilities; adults ≥65 years	397	All stages	Oral hygiene dependence, plaque accumulation, rinsing/gargling ability	FAST	Advanced FAST stages were associated with refusal of oral care, oral hygiene dependence, and plaque accumulation.
Wu (2025) [20]	Taiwan	Cross-sectional observational Study	Taipei Veterans General Hospital; adults aged 60–82 years	81	Mild to Moderate	Tooth loss, Eichner index, DMFT, masticatory performance	MMSE, MoCA	Tooth loss and reduced occlusal support were associated with worse cognitive status and lower MoCA scores.
† Yang (2021) [21]	China	Cross-sectional observational study	Chongqing Medical University communities; adults aged 65–90 years	106	AD, MCI, SCD groups	DMFT, plaque index, attachment loss, GOHAI, salivary microbiota	NPI, MMSE, CDR	Plaque index, attachment loss, and oral health stressors were associated with neuropsychiatric symptoms; perceived stress mediated these relationships.

Abbreviations: AD, Alzheimer’s disease; ADCS-ADL, Alzheimer’s Disease Cooperative Study–Activities of Daily Living; ADL, activities of daily living; BOHSE, Brief Oral Health Status Examination; BOP, bleeding on probing; CAL, clinical attachment loss; CDR, Clinical Dementia Rating; CPI, Community Periodontal Index; CSF, cerebrospinal fluid; DMFT/DMFS, decayed, missing, and filled teeth/surfaces; FAST, Functional Assessment Staging Tool; GDS, Global Deterioration Scale; GOHAI, Geriatric Oral Health Assessment Index; ICD, International Classification of Diseases; MCI, mild cognitive impairment; MMSE, Mini-Mental State Examination; MoCA, Montreal Cognitive Assessment; NHAS, Nursing Home Adjustment Scale; NPI, Neuropsychiatric Inventory; PPD, probing pocket depth; RR, risk ratio; SCD, subjective cognitive decline. Note: † Study included broader cognitive continua, mixed dementia populations, cognitively healthy controls, or non-AD comparator groups. * Study included a control or comparator cohort.

**Table 4:** Oral health findings and cognitive associations.

Author (Year)	Oral Health Exposure or Intervention	Oral Outcomes	Cognitive or Dementia-Related Measures	Key Findings
Aragon (2018) [6]	Oral health assessment	Tooth loss, periodontal disease, candidiasis, low salivary flow	FAST, CDR, GDS	AD associated with significantly poorer oral health than controls
Campos (2018) [15]	Prosthodontic rehabilitation	Improved mastication and oral quality of life	MMSE, CDR	Denture rehabilitation improved oral function in mild AD
Chen (2022) [16]	Structured oral health intervention	Improved BOHSE and microbiota profile	MMSE, NPI, ADCS-ADL	Oral health intervention associated with slower cognitive and functional decline
Frota (2016) [17]	Oral examination and prosthesis assessment	High prevalence of periodontal disease, caries, maladaptive dentures	MMSE diagnostic classification	Dementia patients demonstrated poorer prosthetic and oral conditions
Kulkarni (2023) [13]	Poor oral health cohort comparison	Periodontal disease, tooth loss, caries	ICD-coded AD diagnosis	Poor oral health associated with increased AD risk
Laugisch (2021) [18]	Periodontal assessment	Universal periodontitis across dementia cohorts	MMSE, CSF biomarkers	Periodontal disease prevalent in both AD and non-AD dementia
Liu (2019) [2]	Salivary microbiome profiling	Reduced diversity and altered taxa	MMSE, CDR, ADL	Altered oral microbiome associated with AD status
Sherbaf (2025) [14]	Periodontal evaluation	Increased plaque, probing depth, attachment loss	MMSE	Poorer periodontal status associated with AD severity
Shirobe (2023) [19]	Oral hygiene function assessment	Plaque accumulation, oral care dependence	FAST staging	More severe dementia associated with greater oral hygiene impairment
Wu (2025) [20]	Dental and occlusal assessment	Tooth loss, impaired mastication, reduced occlusal support	MMSE, MoCA	Reduced occlusal support associated with worse cognition
Yang (2022) [21]	Oral health stressor evaluation	Poor oral hygiene, attachment loss, dysbiosis	NPI, perceived stress	Oral stressors directly and indirectly associated with neuropsychiatric symptoms

Abbreviations: AD, Alzheimer’s disease; ADCS-ADL, Alzheimer’s Disease Cooperative Study–Activities of Daily Living; ADL, activities of daily living; BOHSE, Brief Oral Health Status Examination; CDR, Clinical Dementia Rating; FAST, Functional Assessment Staging Tool; GDS, Global Deterioration Scale; ICD, International Classification of Diseases; MMSE, Mini-Mental State Examination; MoCA, Montreal Cognitive Assessment; NPI, Neuropsychiatric Inventory.

**Table 5:** Implementation barriers, care considerations, and mechanistic domains.

Author (Year)	Mechanistic or Implementation Domain	Key Findings	Clinical Implications
Aragon (2018) [6]	Caregiver-supported oral hygiene	Oral hygiene worsened with disease progression	Early preventive dental care may be beneficial
Campos (2018) [15]	Prosthodontic feasibility	Denture rehabilitation tolerated in mild AD	Oral rehabilitation was feasible in selected patients
Chen (2022) [16]	Caregiver-supported intervention	Structured oral care was feasible in long-term care	Multicomponent oral care programs may support cognition
Frota (2016) [17]	Access to dental care and prosthetic maintenance	High prevalence of maladaptive dentures	Need for regular prosthetic monitoring in dementia
Kulkarni (2023) [13]	Population-level epidemiology	Large-scale EHR data supported an oral-systemic association	Supports oral health as a potential modifiable AD risk factor
Laugisch (2021) [18]	Systemic inflammation and periodontitis	Periodontal inflammation common across dementia groups	Reinforces the inflammatory mouth–brain axis hypothesis
Liu (2019) [2]	Oral microbiome dysbiosis	Altered microbial diversity and taxa in AD	Supports microbiome contribution to AD pathophysiology
Sherbaf (2025) [14]	Oral hygiene maintenance barriers	Behavioral resistance and hygiene difficulties common	Caregiver training may improve oral care adherence
Shirobe (2023) [19]	Functional dependence in oral care	Refusal of care and oral hygiene dependence increased with FAST stage	Oral hygiene strategies should be tailored to dementia severity
Wu (2025) [20]	Functional oral decline	Mastication and occlusal support linked to cognition	Functional oral rehabilitation may warrant further study
Yang (2022) [21]	Stress-mediated oral–brain pathway	Perceived stress mediates oral health and neuropsychiatric symptom relationships	Oral health may influence behavioral symptoms through psychosocial mechanisms

Abbreviations: AD, Alzheimer’s disease; EHR, electronic health record; FAST, Functional Assessment Staging Tool.

## Data Availability

The data for this study are contained within the manuscript.

## References

[1] Rostagno A.A. (2023). Pathogenesis of Alzheimer’s disease. Int. J. Mol. Sci..

[2] Liu X.-X., Jiao B., Liao X.-X., Guo L.-N., Yuan Z.-H., Wang X., Xiao X.-W., Zhang X.-Y., Tang B.-S., Shen L. (2019). Analysis of salivary microbiome in patients with Alzheimer’s disease. J. Alzheimer’s Dis..

[3] Leira Y., Domínguez C., Seoane J., Seoane-Romero J., Pías-Peleteiro J.M., Takkouche B., Blanco J., Aldrey J.M. (2017). Is periodontal disease associated with Alzheimer’s disease? A systematic review with meta-analysis. Neuroepidemiology.

[4] Chen C.K., Wu Y.T., Chang Y.C. (2017). Association between chronic periodontitis and the risk of Alzheimer’s disease: A retrospective, population-based, matched cohort study. Alzheimer’s Res. Ther..

[5] Dominy S.S., Lynch C., Ermini F., Benedyk M., Marczyk A., Konradi A., Nguyen M., Haditsch U., Raha D., Griffin C. (2019). *Porphyromonas gingivalis* in Alzheimer’s disease brains: Evidence for disease causation and treatment with small-molecule inhibitors. Sci. Adv..

[6] Aragón F., Zea-Sevilla M.A., Montero J., Sancho P., Corral R., Tejedor C., Frades-Payo B., Paredes-Gallardo V., Albaladejo A. (2018). Oral health in Alzheimer’s disease: A multicenter case-control study. Clin. Oral Investig..

[7] Matsubara C., Shirobe M., Furuya J., Watanabe Y., Motokawa K., Edahiro A., Ohara Y., Awata S., Kim H., Fujiwara Y. (2021). Effect of oral health intervention on cognitive decline in community-dwelling older adults: A randomized controlled trial. Arch. Gerontol. Geriatr..

[8] Guo H., Wang Z., Chu C.H., Chan A.K.Y., Lo E.C.M., Jiang C.M. (2024). Effects of oral health interventions on cognition of people with dementia: A systematic review with meta-analysis. BMC Oral Health.

[9] Hernández-Vásquez A., Barrenechea-Pulache A., Aguirre-Ipenza R., Comandé D., Azañedo D. (2022). Interventions to improve the oral hygiene of individuals with Alzheimer’s disease: A systematic review. Dent. J..

[10] Aida J., Kiuchi S., Shirai K., Peres M.A., Matsuyama Y. (2026). Oral health and dementia: Causal inference and theoretical mechanisms. J. Dent. Res..

[11] Arksey H., O’Malley L. (2005). Scoping studies: Towards a methodological framework. Int. J. Soc. Res. Methodol..

[12] Tricco A.C., Lillie E., Zarin W., O’Brien K.K., Colquhoun H., Levac D., Moher D., Peters M.D.J., Horsley T., Weeks L. (2018). PRISMA extension for scoping reviews (PRISMA-ScR): Checklist and explanation. Ann. Intern. Med..

[13] Kulkarni M.S., Miller B.C., Mahani M., Mhaskar R., Tsalatsanis A., Jain S., Yadav H. (2023). Poor oral health linked with higher risk of Alzheimer’s disease. Brain Sci..

[14] Sherbaf R.A., Kaposvári G.M., Nagy K., Pakáski M., Gajdács M., Matusovits D., Baráth Z. (2025). Oral health status and factors associated with oral health in patients with Alzheimer’s disease: A matched case-control observational study. J. Clin. Med..

[15] Campos C.H., Ribeiro G.R., Rodrigues Garcia R.C.M. (2018). Mastication and oral health-related quality of life in removable denture wearers with Alzheimer disease. J. Prosthet. Dent..

[16] Chen L., Cao H., Wu X., Xu X., Ji X., Wang B., Zhang P., Li H. (2022). Effects of oral health intervention strategies on cognition and microbiota alterations in patients with mild Alzheimer’s disease: A randomized controlled trial. Geriatr. Nurs..

[17] Frota B.M.D., Holanda S.N., Sousa F.B., Alves A.P.N.N. (2016). Evaluation of oral conditions in patients with neurodegenerative diseases treated in geriatric centers. Rev. Gaucha Odontol..

[18] Laugisch O., Johnen A., Buergin W., Eick S., Ehmke B., Duning T., Sculean A. (2021). Oral and periodontal health in patients with Alzheimer’s disease and other forms of dementia: A cross-sectional pilot study. Oral Health Prev. Dent..

[19] Shirobe M., Edahiro A., Motokawa K., Morishita S., Ohara Y., Motohashi Y., Iwasaki M., Watanabe Y., Hirano H. (2023). Association between dementia severity and oral hygiene management issues in older adults with Alzheimer’s disease: A cross-sectional study. Int. J. Environ. Res. Public Health.

[20] Wu S.-Y., Wu C.-Y., Lin Y.-S., Lin C.-S., Lee W.-J., He S.-J., Lin G.-H., Huang H.-Y., Fuh J.-L. (2025). Oral health variables associated with factors across the Alzheimer’s disease continuum: From subjective cognitive decline to dementia. J. Dent..

[21] Yang B., Tao B., Yin Q., Chai Z., Xu L., Zhao Q., Wang J. (2021). Associations between oral health status, perceived stress, and neuropsychiatric symptoms among community individuals with Alzheimer’s disease: A mediation analysis. Front. Aging Neurosci..

[22] Yi Y., Lee C.H., Shin H.S., Shin S. (2025). Oral diseases as emerging risk factors for alzheimer’s disease: A scoping review. Jpn. Dent. Sci. Rev..

[23] Felicetti A., Azzolino D., Piro P.P., Lopes G.C.D., Rezaeinezhad N., Lovero R., Bocchio-Chiavetto L., Colella M., Passarelli P.C. (2025). The oral-brain axis in alzheimer’s disease: From microbial dysbiosis to neurodegeneration. Microorganisms.

